# Enhanced antitumor immunity through sequential targeting of PI3Kδ and LAG3

**DOI:** 10.1136/jitc-2020-000693

**Published:** 2020-10-22

**Authors:** Sarah Nicol Lauder, Kathryn Smart, Veerle Kersemans, Danny Allen, Jake Scott, Ana Pires, Stefan Milutinovic, Michelle Somerville, Sean Smart, Paul Kinchesh, Elena Lopez-Guadamillas, Ellyn Hughes, Emma Jones, Martin Scurr, Andrew Godkin, Lori S Friedman, Bart Vanhaesebroeck, Awen Gallimore

**Affiliations:** 1 Infection and Immunity, Cardiff University Department of Medicine, Cardiff, UK; 2 Infection and Immunity, University of Oxford, Oxford, UK; 3 CRUK/MRC Oxford Institute for Radiation Oncology, Department of Oncology, University of Oxford, Oxford, UK; 4 UCL Cancer Institute, Paul O'Gorman Building, University College London, London, UK; 5 Cancer Biomarker Group, Cancer Research UK Manchester Institute, The University of Manchester, Manchester, UK; 6 ORIC Pharmaceuticals, South San Francisco, California, USA

**Keywords:** lymphocytes, tumor-infiltrating, T-lymphocytes, immunotherapy

## Abstract

**Background:**

Despite striking successes, immunotherapies aimed at increasing cancer-specific T cell responses are unsuccessful in most patients with cancer. Inactivating regulatory T cells (Treg) by inhibiting the PI3Kδ signaling enzyme has shown promise in preclinical models of tumor immunity and is currently being tested in early phase clinical trials in solid tumors.

**Methods:**

Mice bearing 4T1 mammary tumors were orally administered a PI3Kδ inhibitor (PI-3065) daily and tumor growth, survival and T cell infiltrate were analyzed in the tumor microenvironment. A second treatment schedule comprised PI3Kδ inhibitor with anti-LAG3 antibodies administered sequentially 10 days later.

**Results:**

As observed in human immunotherapy trials with other agents, immunomodulation by PI3Kδ-blockade led to 4T1 tumor regressor and non-regressor mice. Tumor infiltrating T cells in regressors were metabolically fitter than those in non-regressors, with significant enrichments of antigen-specific CD8^+^ T cells, T cell factor 1 (TCF1)^+^ T cells and CD69^−^ T cells, compatible with induction of a sustained tumor-specific T cell response. Treg numbers were significantly reduced in both regressor and non-regressor tumors compared with untreated tumors. The remaining Treg in non-regressor tumors were however significantly enriched with cells expressing the coinhibitory receptor LAG3, compared with Treg in regressor and untreated tumors. This striking difference prompted us to sequentially block PI3Kδ and LAG3. This combination enabled successful therapy of all mice, demonstrating the functional importance of LAG3 in non-regression of tumors on PI3Kδ inhibition therapy. Follow-up studies, performed using additional cancer cell lines, namely MC38 and CT26, indicated that a partial initial response to PI3Kδ inhibition is an essential prerequisite to a sequential therapeutic benefit of anti-LAG3 antibodies.

**Conclusions:**

These data indicate that LAG3 is a key bottleneck to successful PI3Kδ-targeted immunotherapy and provide a rationale for combining PI3Kδ/LAG3 blockade in future clinical studies.

## Introduction

The efficacy of a tumor-specific immune response is influenced by the balance between tumor-infiltrating regulatory T cells (Treg) and tumor infiltrating CD4^+^ and CD8^+^ T cells (reviewed in Takeuchi and Nishikawa^1^). Strategies in mice and humans have sought to deplete Treg, with some success,^2–5^ supporting the drive to develop more effective, clinically relevant Treg-targeting approaches. Mouse and human studies have demonstrated that genetic or pharmacological inactivation of the phosphoinositide 3-kinase δ (PI3Kδ) isoform of PI3K has the capacity to reduce the number of Treg and dampen their function.^6–8^

The PI3K family members that are directly involved in cell signaling comprise PI3Kα, PI3Kβ, PI3Kδ and PI3Kγ, of which PI3Kδ is expressed primarily by leukocytes.^9^ Small molecule inhibitors targeting single and multiple isoforms of PI3K have been developed, including the first approved PI3K inhibitor idelalisib (formerly GS-1101, CAL-101), a PI3Kδ-selective inhibitor licensed for the therapy of specific B cell lymphomas.^10 11^ While in the case of these hematological cancers the PI3Kδ inhibitors are thought to target the cancer cells directly, recent studies have demonstrated that inhibiting PI3Kδ additionally promotes antitumor immunity in both hematological and solid cancers, through its preferential dampening of Treg.^6 12–15^ Previous studies have demonstrated that germline genetic inactivation of PI3Kδ in mice results in T cell-mediated control of tumor growth.^6^ Similarly, treating mice with a pharmacological inhibitor of PI3Kδ is associated with improved control of tumor growth.^6^

PI3K blockade has recently been shown to affect the discordant transmission of PI3K in dividing T cells.^16 17^ This asymmetrical localization of PI3K enables activated T cells to give rise to daughter cells with either an effector or self-renewing phenotype.^17^ Interestingly, the latter T cell type has been shown in many studies to exhibit the greatest antitumor capacity both in mouse models and in patients with cancer.^18–20^ It is reasonable therefore to hypothesize that, through interfering with this process, PI3Kδ blockade biases cell fate in a manner that is favorable to the development of robust tumor-specific T cell responses.

Using a mouse model of triple negative breast cancer (4T1 cell line) which exhibits the hallmarks of aggressive human disease,^21^ Ali and colleagues previously showed that Treg activity is preferentially dampened over other T cell subsets on systemic PI3Kδ blockade,^6^ resulting in tumor immunity. We initially repeated these experiments with PI3Kδ-blockade and demonstrated that mice can be divided into non-regressors (where tumor growth rate is reduced but tumors continue to grow) and regressors (where tumors shrink). We used these two groups (regressors and non-regressors) to examine the nature of Tregs and tumor-specific effector T cells within the tumors. This not only allowed us to identify correlates of successful tumor immunity, but conversely the key bottleneck preventing an effective immune response, and in particular, the crucial role of the inhibitory receptor LAG3 in controlling this balance.

## Materials and methods

### Mice and cell lines

Female BALB/c and C57BL/6 mice were purchased from Charles River and housed in filter-top cages in specific pathogen-free conditions, with standard chow and water provided ad libitum. Experiments were conducted in accordance with Home Office UK guidelines. The tumor cell lines 4T1 (obtained from ATCC (CRL-2539), CT26 (obtained from ATCC (CRL-2638)) and MC-38 (gift from Prof David Withers, University of Birmingham, UK) were maintained in culture medium (RPMI 1640, 10% FCS, 2 mM L-glutamine, 1 mM sodium pyruvate and 50 mg/mL penicillin-streptomycin). 1×10^5^ 4T1 cells were injected subcutaneous into the mammary fat pad, 5×10^5^ CT26 or MC-38 cells were injected subcutaneously into the flank. Tumors were measured from day 7 up to three times per week until the mouse was sacrificed and tumor measured using digital calipers. The following calculation was used to determine tumor volume: (length×width×short)×(3.14/6) (where short equals the lower of the length and width measurements and provides an estimate of height). Tumor weight was measured at endpoint following excision of the tumor from the host.

### In vivo drug treatment

PI-3065 was provided by Genentech and administered by daily oral gavage at 75 mg/kg as described in the original paper of Ali and colleagues.^6^ This dosing of PI-3065 was subsequently validated by others to provide selectivity for PI3Kδ.^22^ Vehicle treated mice were given an equivalent volume of carrier solution. Mice were dosed daily either from day −1 prior to tumor inoculation or from day 7 after tumor inoculation until the termination of the experiment. For CD8^+^ T cell depletion studies, mice were administered 400 µg of a CD8-depleting antibody (clone YTS169.4) on day 6, 8 and 15 post-tumor inoculation. For combination therapy studies, mice were administered 100 µg of anti-CD69 antibody (clone H1.2F3, Thermo-Fisher) or 250 µg of anti-LAG3 antibody (clone C9B7W, BioXCell) three times per week from day 10.

### Sample dissociation

Tumors were isolated from tumor bearing mice, mechanically disaggregated and then incubated at 37°C for 30 min in tumor dissociation mix (HBSS, 10% FCS, 2 mg/mL collagenase, 0.03 mg/mL DNAse I). Following incubation tumor cells were passed through a 40 µm filter and washed two times in RPMI supplemented with 10% FCS, to yield a single cell suspension.

### Flow cytometry

Cells were washed twice with PBS and stained using LIVE/DEAD Aqua (Invitrogen) according to the manufacturer’s instructions. Cells were washed two times with FACS buffer and Fc receptors blocked with anti-CD16/32 (clone 93; Biolegend). Cells were surface stained with the following antibodies: CD3 (Biolegend, 17A2, BV785 or PECy7), CD4 (Biolegend, GK1.5, BV605, APC, FITC or PE), CD8 (Biolegend, 53–6.7, BV421, APC/Fire 750), CD45 (Biolegend, 30-F11, PECy7), CD69 (Biolegend, H1.2F3, PECy7), PD1 (Biolegend, 29F.1.A12, BV421), LAG3 (Biolegend, C9B7W, PE). For intracellular and transcription factor staining cells were processed using a Foxp3/Transcription factor staining buffer set (eBioscience) and stained with antibodies, FoxP3 (eBioscience, FJK-16s, APC), Ki67 (Biolegend, 11F6, AF488), Glut1 (Abcam, EPR3915, AF488), TCF1/7 (Cell Signalling, 6790S, AF647). To enumerate antigen-specific T cells the PE-labeled dextramer (H-2L^d^ SPSYVYHQ) was used according to the manufacturer’s instructions. Mitochondrial membrane potential was determined using Mitospy-Orange (Biolegend) according to manufacturer’s instructions. Cell numbers were compared in tumors of individual mice at the same time-points after tumor cell inoculation; numbers were therefore normalized per gram of tumor tissue.

### Dynamic contrast enhanced MRI

Mice were anesthetized and maintained at a body temperature of 35°C using an MRI compatible electrical heating system^23^ and a respiration rate of 50–60 breaths per min. Dynamic contrast enhanced MRI (DCE-MRI) was performed at 7.0 T (VNMRS, Varian) using a 25 mm diameter, 35 mm long quadrature birdcage RF coil (Rapid Biomedical) operated in transmit/receive mode. Baseline T1 was measured using a variable flip angle implementation of a respiratory-gated constant TR 3D gradient echo scan (TE=0.64 ms TR=1.60 ms, FA=1–8 deg, isotropic resolution of 420 µm), which incorporated dynamic reacquisition of data corrupted on entry to each breath.^24^ B1 transmission inhomogeneities were corrected using a similarly gated Actual Flip Angle Imaging scan. DCE was performed by repeating the three-dimensional gradient echo scan 50 times, at FA=5 deg, with Gd contrast agent (Omniscan, 30 µL) infused automatically by syringe pump (PHD2000, Harvard Apparatus) over 5 s starting at the beginning of scan 11/50.

### Immunohistochemical staining

Tumors were excised and placed into 10% neutral-buffered formalin saline. Fixed tumors were embedded into paraffin and 5 µm sections were cut from wild-type and mice which carry a point mutation in the ATP-binding site of PI3Kδ which renders this kinase inactive (δ^D910A^ mice^25^) and from vehicle-treated and PI-3065-treated mice. Sections were stained for high endothelial venules (HEV) as described previously.^26^ Briefly, antigen retrieval was performed in Tris-EDTA (10 mmol/L; 1 mmol/L, pH 9.0). Endogenous peroxidase activity was quenched using 1% H_2_O_2_/MeOH and non-specific binding was blocked with 2.5% horse serum. Tumor sections were incubated overnight at 4°C with rat peripheral node addressin (PNAd) (clone MECA79, Biolegend). Sections were then washed and incubated in anti-Rat ImmPRESS HRP polymer detection reagent (VectorLabs). Slides were incubated briefly in Vector Chromagen DAB HRP substrate (Vector Laboratories). Slides were then rinsed with dH_2_O and then counterstained with hematoxylin and mounted in DPX. Sections were imaged at 40× magnification using a Zeiss Axio Scan.Z1 slide scanner (Plan-Apochromat 40×/0.95 Korr M27). The total HEV area within each tumor was then calculated using Zen software.^2 27^

### In vitro T cell activation assays

CD8^+^ T cells were purified from splenocytes derived from naïve BALB/c mice using negative magnetic bead selection kits (Biolegend). CD8^+^ T cells were labeled with Tag-It-Violet (Biolegend) prior to stimulation with 1 µg/mL CD3/CD28 activator beads (Dynabeads) and 30 IU/mL IL-2. Cells were left untreated, or treated with vehicle (Dimethyl sulfoxide) or 10 µM PI-3065 for 5 days prior to flow cytometric analysis.

### Statistics

Statistical analysis was performed using Graphpad Prism V.8. Unless stated otherwise in the figure legends, data are displayed as the mean±SEM. Statistical significance is denoted as follows: *p<0.05; **p<0.01; ***p<0.001.

## Results

### Regressor and non-regressor tumors in mice treated with a PI3Kδ inhibitor

**Figure 1:**
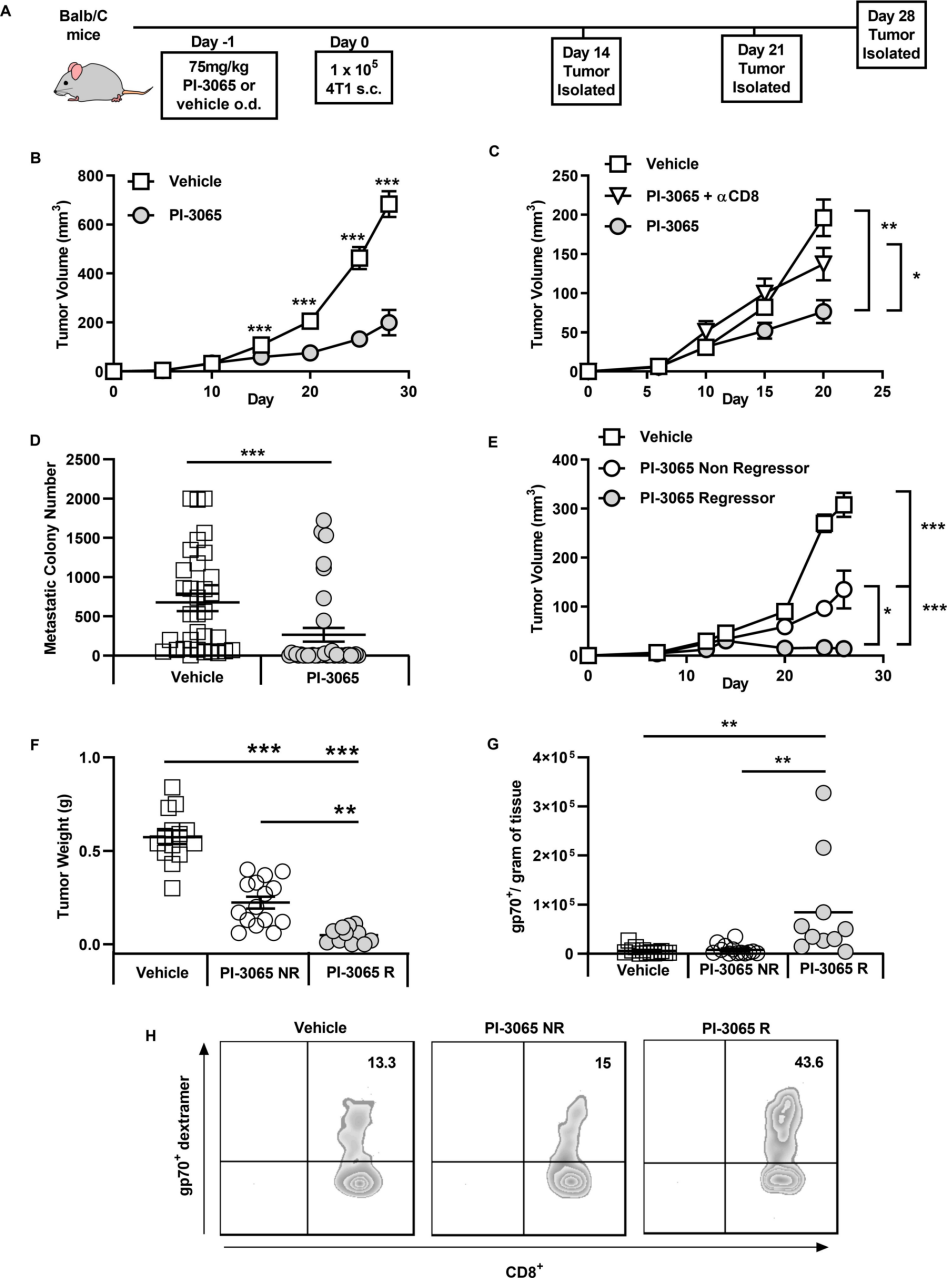
Inhibition of PI3Kδ controls 4T1 tumor growth and is dependent on CD8 T cells. (A) Schematic diagram shows the experimental protocol: BALB/c mice were treated daily for the duration of the study with either 75 mg/kg of PI-3065 or vehicle by oral gavage. One day later mice were inoculated with tumor cells. Tumors were harvested at the time points indicated. (B) Tumor growth curves from mice inoculated with 4T1 cells (8–64 mice/group/time-point). (C) Tumor growth curves of mice treated with PI-3065 (daily) and anti-CD8 depleting antibody at day 6, 8 and 15 post-tumor inoculation (8 mice/group/time point). (D) Number of metastatic colonies in the lungs of tumor bearing mice at day 28 post-tumor inoculation (33–38 mice/group). (E) Tumor growth curves and (F) tumor burden from mice treated with PI-3065 who regress their tumor (PI-3065 regressor) or mice treated with PI-3065 who slow their tumor growth rate (PI-3065 non-regressor) (11–15 mice/group). (G) Number of gp70 dextramer^+^ CD8^+^ T cells per gram of tumor tissue in vehicle-treated mice, PI-3065 non-regressors and PI-3065 regressors. (H) Representative histograms of gp70^+^ dextramer staining. All data are displayed as the mean±SEM statistical significance was determined by multiple unpaired t-test (B, C, E), Mann-Whitney test (D), one-way Analysis of Variance (ANOVA) (F) or Kruskal-Wallis test (G). (*p≤0.05, **p≤0.01, ***p≤0.001).

It has been previously demonstrated that inactivation of PI3Kδ in mice can significantly reduce the growth rate of inoculated solid tumors.^6^ We examined the tumor growth rates of mice treated daily with either PI-3065 (PI3Kδ inhibitor) or vehicle (dosing control) in the 4T1 breast tumor model (figure 1). Confirming previous studies, we observed that treatment with PI-3065 resulted in a significant reduction in tumor growth rate in the 4T1 model, (figure 1B). We observed that treatment with PI-3065 led to CD8^+^ T cell-dependent control of primary tumor growth (figure 1C) and reduced metastatic burden (figure 1D). Overall, these data demonstrate a direct or indirect effect of PI-3065 on the ability of CD8^+^ T cells to mount an effective antitumor response.

While tumors from most treated mice exhibited reduced growth rates, we found that mice could be divided into non-regressors (where tumor growth rate was reduced but tumors continued to grow) and regressors (where tumors shrank, approximately 12.5% of the mice) (figure 1E, F). When we examined the expansion of tumor-specific CD8^+^ T cells using a tetramer specific for the AH1 epitope of the immunodominant 4T1 antigen gp70 in tumors that could be definitively designated ‘regressor’ or ‘non-regressor’ (day 28), we found that a significant expansion of gp70-reactive CD8^+^ T cells was only observed in regressing tumors (figure 1G, H).

### PI3Kδ inhibition results in accumulation of tumor-infiltrating lymphocytes with superior self-renewal capacity

**Figure 2:**
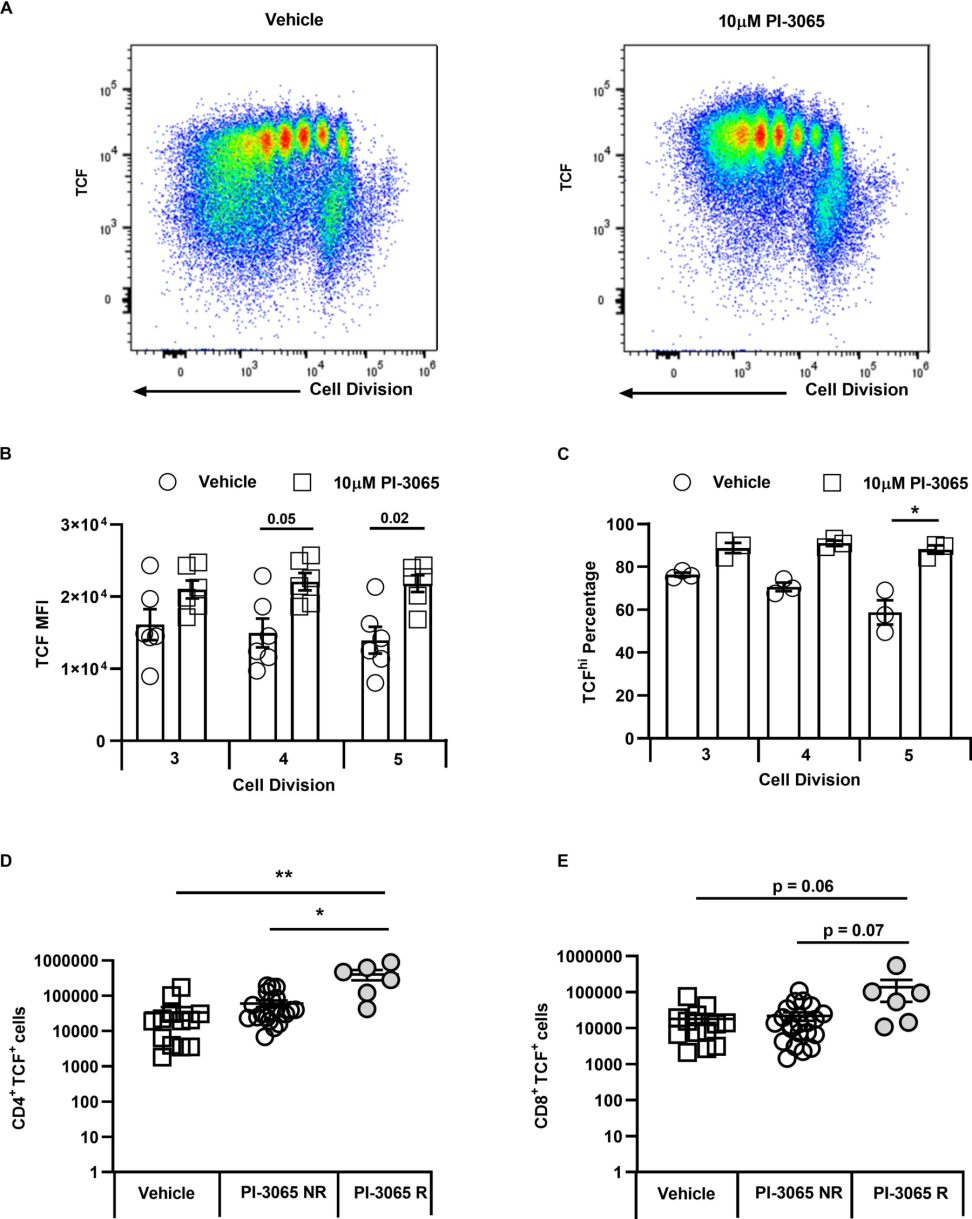
PI3Kδ activity maintains T cell factor (TCF) expression in the 4T1 model. (A) Representative flow cytometry plots showing cell division and TCF1 expression at day 5 in presence of either vehicle or PI-3065. (B) Mean Fluorescence Intensity (MFI) of TCF1 expression on CD8^+^ T cells at division 3, 4 and 5. (C) Percentage of TCFhi CD8^+^ T cells at divisions 3, 4 and 5. (D) Number of tumor infiltrating CD4^+^ T cells and (E) CD8^+^ T cells expressing TCF1 at day 28 post-tumor inoculation normalized to tumor weight. All data are displayed as the mean±SEM, n=5–21 mice/group. Statistical significance was determined by one-way Analysis of Variance (ANOVA) (B, C, D) or Kruskal-Wallis test (E).

To examine the mechanism underpinning enhanced antitumor immunity in PI-3065-treated mice, we first addressed whether this inhibitor directly affected CD8^+^ T cells in vitro. It has been previously shown that after 3–4 rounds of cell division, discordant silencing of TCF1 becomes apparent in sibling cells as a result of unequal transmission of PI3K protein levels between daughter cells.^16 17^ To address whether blockade of PI3Kδ with PI-3065 affected asymmetrical expression of TCF1 in activated T cells, splenic CD8^+^ T cells were labeled with Tag-it-violet and stimulated for 5 days with anti-CD3 and anti-CD28-coupled to beads in the presence or absence of PI-3065. While after 3–4 divisions, TCF1 was, as expected, reduced in a proportion of the control cells, those treated with PI-3065 maintained expression of TCF1 for longer (figure 2A, C), implying that PI3Kδ blockade promotes their self-renewal capacity, possibly also in vivo, thereby promoting tumor immunity.

Since similar tracking of T cell division was not possible in the mouse tumors, we surmised that a bias towards TCF1^+^ cells in PI-3065-treated mice would manifest itself as an accumulation of these cells in treated compared with control tumors. We therefore assessed frequencies of TCF1^+^ cells per gram of tumor tissue in PI-3065-treated versus control mice, including regressor and non-regressor tumors in the analysis (day 28). An increase in TCF1^+^ CD4^+^ and CD8^+^ tumor-infiltrating lymphocyte (TIL) was indeed observed, but only in regressor tumors and significantly so in the case of the CD4^+^ T cell population (figure 2D, E). While it is clear that regressor tumors are smaller than non-regressor tumors at this time-point, it is important to note that a comparison of tumors of equivalent size is not possible due to the general paucity of infiltrating lymphocytes in small tumors at earlier time-points and our inability at these early time-point to distinguish between non-regressor and regressor tumors. Overall, the data described herein, indicate that successful anticancer treatment with a PI3Kδ inhibitor is accompanied by an accumulation of T cells with superior self-renewal capacity.

### PI3Kδ inhibition increases T cell fitness and cell surface expression of Glut1

**Figure 3:**
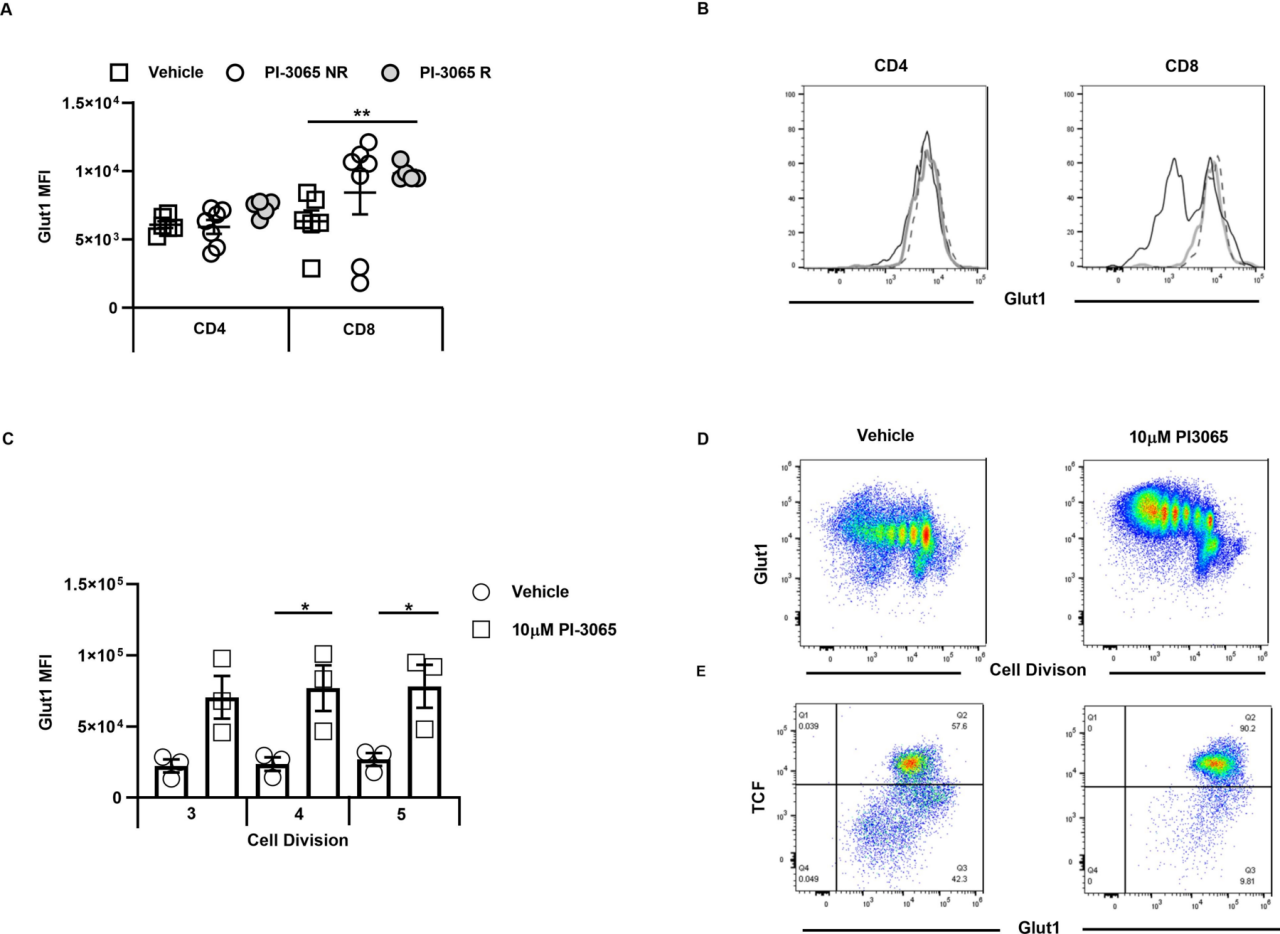
Inhibition of PI3Kδ increases CD8^+^ T cell Glut1 expression in the 4T1 model. Mean Fluorescence Intensity (MFI) of Glut1 expression on (A) CD4^+^ and CD8^+^ tumor infiltrating T cells at day 28 post-tumor inoculation, in vehicle-treated mice and PI-3065 treated non-regressor (NR) or tumor regressors (R). (B) Representative histograms of Glut1 staining of CD4 (left panel) and CD8 (right panel), open black histogram: vehicle-treated, thick gray line: PI-3065 non-regressor, dashed line: PI-3065 regressors. (C) MFI of Glut1 on TCFhi CD8^+^ T cells at divisions 3, 4 and 5. (D) Representative flow cytometry plots showing cell division and Glut1 at day 5 in the presence of vehicle or 10 µM PI-3065. (E) Representative flow cytometry plots showing TCF1 and Glut1 expression in CD8^+^ T cells that have divided five times in the presence of either vehicle or 10 µM PI-3065 (4–7 mice/group). Statistical significance was determined by one-way Analysis of Variance (ANOVA) (A, B, C) (*p≤0.05, **p≤0.01).

During activation and proliferation, T cells use glycolysis to meet their metabolic demands, with the inducible glucose transporter, Glut1, enabling the necessary glucose uptake by T cells. Since TCF1 has been linked to low expression of Glut1,^17^ we wished to examine whether there were differences in the metabolic fitness of TIL recovered from untreated compared with PI-3065-treated mice. We were unable to recover sufficient numbers of CD8^+^ T cells for functional metabolic studies such as oxygen consumption rate, even when pooling cells for groups of tumors. We therefore used flow cytometry to examine whether maintenance of TCF1 after PI3Kδ-blockade impacted Glut1 expression on TILs of PI-3065-treated mice. While no differences were observed between CD4^+^ T cell populations recovered from the different tumor types, Glut1 was clearly higher on CD8^+^ T cells from PI-3065-treated mice, and significantly so on those isolated from regressor tumors (figure 3A, B), implying that the cells may undergo glycolytic metabolism. Similar findings were observed in vitro where cells stimulated for 5 days with bead-bound anti-CD3- and anti-CD28 in the presence of PI-3065 retained TCF1 expression, with increased expression of cell surface Glut1 (figure 3C, D, E). While expression of Glut1 does not necessarily indicate that T cells efficiently take up glucose to support glycolytic metabolism, it is clear, as shown in figure 3A, that high Glut1 on CD8^+^ T cells was significantly associated with regressor tumors. Effector T cells require high rates of glucose metabolism and it is known that improving the metabolic fitness of TIL promotes antitumor responses and successful control of tumor burden (reviewed in O'Sullivan *et al*^28^).

**Figure 4:**
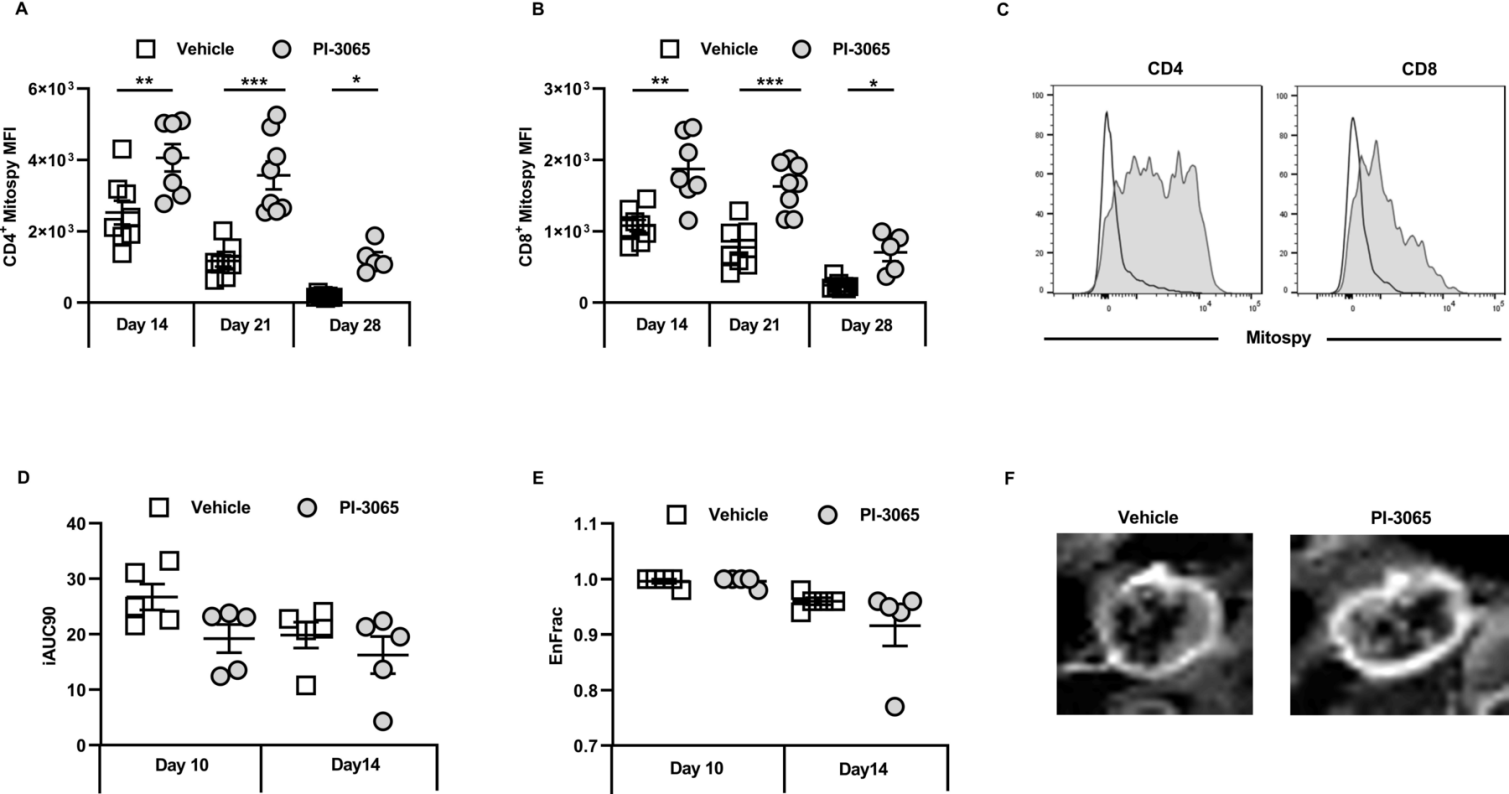
Inhibition of PI3Kδ improves intratumoral T cell fitness in the 4T1 model. MFI of Mitospy expression on tumor infiltrating (A) CD4^+^ T cells and (B) CD8^+^ tumor infiltrating T cells at the indicated day post-tumor inoculation. (C) Representative histograms of Mitospy staining of CD4 (left panel) and CD8 (right panel), open histogram: vehicle treated, shaded histogram: PI-3065 treated. (D) Initial area under the curve determined by dynamic contrast enhanced (DCE)-MRI. (E) The fraction of enhanced voxels during the scan as determined by DCE-MRI. (F) Representative image of a DCE-MRI scan performed at day 14 post-tumor inoculation. The image displays the contrast enhancement after gadolinium infusion (5–8 mice/group). Statistical significance was determined by one-way ANOVA (A) and Kruskal-Wallis test (B) (*p≤0.05, **p≤0.01, ***p≤0.001).

As well as glycolytic insufficiency, defective mitochondrial function has also been linked to impaired antitumor immune responses.^29^ We therefore assessed the mitochondrial membrane potential in TILs as a proxy measure of their energy capacity in treated and control tumors. Staining with Mitospy Orange clearly indicated a significantly increased mitochondrial membrane potential in tumor-infiltrating CD4^+^ and CD8^+^ T cells from treated compared with control mice; this was observed in non-regressor as well as regressor mice (figure 4A, B). Taken together these data demonstrate that inhibiting PI3Kδ confers an enhanced metabolic activity in tumor-infiltrating CD8 T cells that positively correlates with effective control of tumor growth in these mice.

Since it has previously been shown that Glut1 expression increases when T cells are activated under hypoxic conditions,^30^ we measured whether at days 10 and 14, which just precedes deviation into non-regressor and regressor tumors (figure 1), the degree of hypoxia within and/or between groups varied. For this purpose, DCE-MRI was used as a non-invasive, surrogate measure of hypoxia in individual tumors. A reduction in tumor perfusion as measured by the area under the curve (iAUC at 90 s post Gadolinium injection) was observed by day 10 in mice given PI-3065, and both vehicle control and inhibitor-treated mice by day 14 (figure 4D). Enhancement of the fraction of voxels assessed by DCE-MRI was also comparable between the two treatment groups (figure 4E, F). Overall, these data indicate that there was no relationship between the degree of hypoxia and response to treatment.

### Phenotypically distinct effector CD8^+^ T cells are associated with successful PI3Kδ-targeted immunotherapy

**Figure 5:**
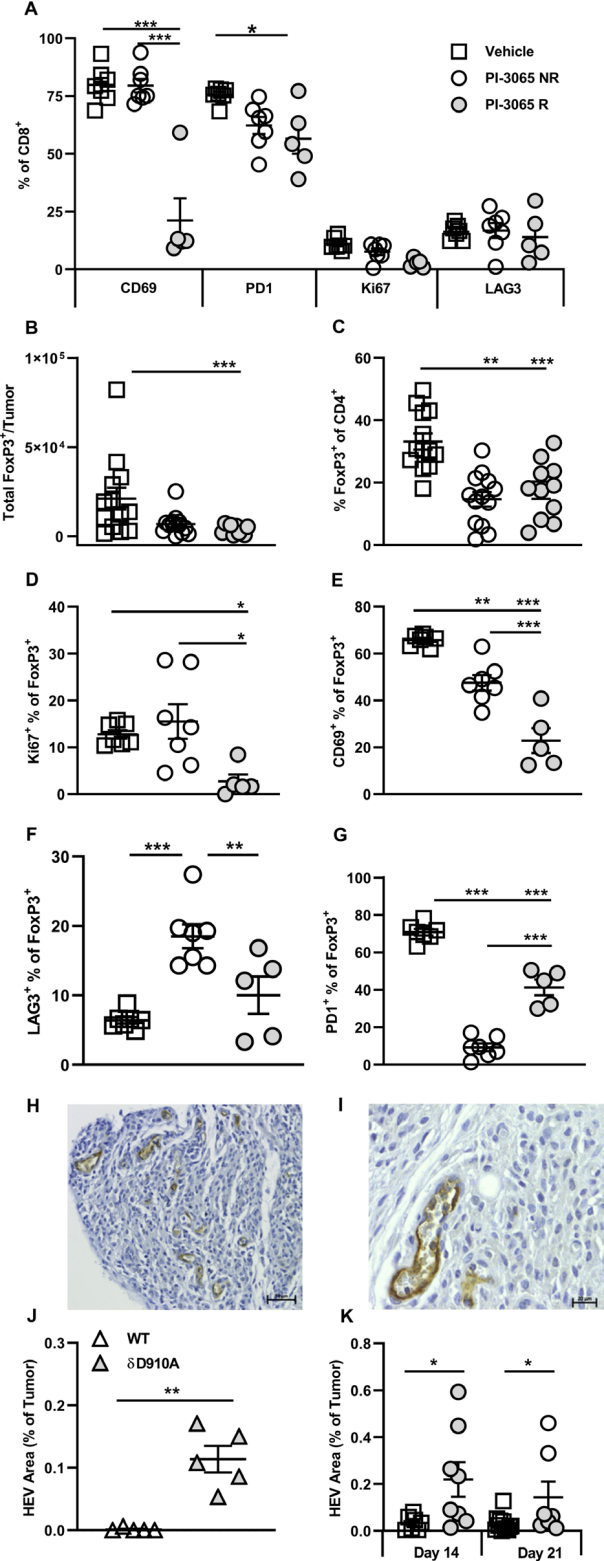
Successful PI3Kδ inhibition in the 4T1 model results in phenotypically distinct CD8^+^ tumoral T cells and Tregs. (A) Percentage expression of T cell phenotypic markers on CD8^+^ tumor infiltrating T cells at day 28 post-tumor inoculation. (B) Total number and (C) percentage of FoxP3^+^ CD4^+^ T cells and present in tumors at day 28 post-tumor inoculation in the three treatment groups. Percentage of FoxP3^+^ CD4^+^ T cells expressing Ki67 (D), CD69 (E), LAG3 (F) and PD1 (G) at day 28 post-tumor inoculation in the three treatment groups. (H and I) Representative high endothelial venules (HEV) staining in 4T1 tumors. (J) Percentage HEV area in tumors from wild-type and PI3Kδ kinase dead mice (δD910A) at day 21 post-tumor inoculation. (K) Percentage HEV area in tumors from mice in the three treatment groups, squares: vehicle treated, shaded circles: PI-3065 treated, open circles: PI-3065 treated regressor. All data are displayed as the mean±SEM, N=5–12 mice/group (NR: non-regressor, R; regressor). Statistical significance was determined by one-way ANOVA (A, C, E, G), Kruskal-Wallis test (B, D) or unpaired T test (J and K). (*p≤0.05, **p≤0.01, ***p≤0.001).

Sustained signaling is linked to exhaustion of CD8^+^ T cells, characterized by upregulation of inhibitory receptors such as PD1 and LAG3. T cells expressing either or both of these receptors are often highly enriched within the TILs of many different cancer types (reviewed in Nguyen and Ohashi^31 32^), and we have also observed this in human colon cancer.^33^ We hypothesized that interfering with PI3K signaling by in vivo blockade of PI3Kδ, would prevent or slow down exhaustion of TILs. To test this, CD8^+^ TILs in control, non-regressor and regressor tumors were compared for expression of PD1 and LAG3 (figure 5A). While the proportion of LAG3^+^CD8^+^ T cells was low in all tumor types, no significant differences were observed after treatment in both non-regressor and regressor tumors. The patterns of PD1 expression, however, were altered, with a lower proportion of CD8^+^ T cells expressing PD1 in treated compared with control mice; a difference which reached significance in regressor tumors (figure 5A). This finding implies that through attenuating TCR signaling and/or promoting the accumulation of self-renewing T cells, CD8 T cell exhaustion is restrained on PI3Kδ blockade.

Since it has been reported that tumors grow more slowly in mice lacking the early activation marker CD69 (a transmembrane C-Type lectin) or in mice treated with CD69-specific antibodies,^34 35^ we next compared expression of CD69 on TILs in control and treated tumors. A highly significant reduction in the proportion of CD69^+^ CD8^+^ T cells in regressor compared with both control and non-regressor tumors was observed (figure 5A). Overall, from this phenotypic analysis, lack of CD69 expression emerged as the major feature distinguishing CD8^+^ TILs in regressor tumors, implying that it serves as a key checkpoint for successful PI3Kδ-targeted immunotherapy.

### Phenotypically distinct Treg are associated with successful PI3Kδ-targeted immunotherapy

The previous study by Ali and colleagues showed that mice with genetically inactivated PI3Kδ or treated with PI-3065, exhibited Treg reduced in number and functional capacity.^6^ Moreover, the study clearly showed that Treg-dysfunction was linked to enhanced antitumor immunity.^6^ We sought to address the impact of PI-3065 on Treg in regressor versus non-regressor tumors. PI-3065 treatment significantly reduced both the total number of Treg and the proportion of FoxP3^+^ T cells in TILs (figure 5B, C) but neither the number nor proportion of Treg distinguished regressor from non-regressor tumors.

Using a different mouse tumor model whereby transgenic Treg were depleted with diphtheria toxin, we previously showed that a successful antitumor T cell response was associated with the neogenesis of intratumoral HEV.^2 27^ These studies also showed that T cells, activated as a result of Treg-depletion, drove neogenesis of intratumoral HEV.^2^ We sought to determine whether inactivation of Treg through inhibiting PI3Kδ would have a similar effect. We first tested whether this might be the case using tumors generated in wild-type (WT) mice and PI3Kδ-inactive (PI3Kδ^D910A^) mice described previously.^13^ The HEV phenotype is characterized by expression of PNAd with coexpression of the pan-endothelial cell marker, CD31 and a plump and cuboidal morphology (figure 5H, I).^26^ Vessels displaying this phenotype, thus confirming the presence of HEV, were detected in 18% and 89% of 4T1 tumors in WT and PI3Kδ^D910A^ mice, respectively. The average area of HEVs was calculated (μm^2^) per tumor in these (figure 5J) and in PI-3065-treated mice (figure 5K). In the case of the latter, tumor tissue collected at days 14 (before regressor and non-regressors can be distinguished) and at day 21 (allowing regressors and non-regressors to be distinguished) was stained for HEV markers. HEV area was measured and found to be higher in PI-3065-treated compared with control tumors, particularly in regressor tumors. This result extends our previous findings in Foxp3-DTR transgenic mice to show that neogenesis of intratumoral HEV is observed following administration of a therapeutically relevant inhibitor of Tregs.^2 27^

As an additional measure of Treg function, we compared Treg in control, non-regressor and regressor tumors for expression of PD1 and LAG3 (figure 5F, G).^36^ The pattern of expression within and between tumor types was noteworthy in so much that a high proportion of PD1^+^ cells corresponded with a low proportion of LAG3^+^ cells and vice versa, implying a degree of compensatory expression by the two receptors. The proportion of Treg expressing PD1 was significantly reduced in treated tumors, compatible with a reduced function of Treg in these tumors. This does not account for tumor regression versus non-regression however, as the proportion of PD1^+^ Treg was in fact, higher in regressor tumors. On the other hand, the proportion of Treg expressing LAG3 was significantly higher in non-regressors compared with both control and regressor tumors, implying that LAG3 plays a role in maintaining the suppressive function of the remaining Treg in non-regressor tumors.^36^

Finally, since we have previously shown that expression of the early activation marker CD69 denotes intratumoral Treg with superior suppressive capacity,^37^ we compared CD69 expression in control, non-regressor and regressor tumors. We found that Treg remaining in regressor tumors were significantly less likely to express CD69 than those in control and non-regressor tumors (figure 5E). Similarly, proliferation, as measured by Ki67 staining, was also reduced among Treg in regressor tumors (figure 5D). These data point to the presence of poorly proliferating, dysfunctional Treg in regressor tumors whereas the remaining Treg in non-regressor tumors may still exert suppressive effects.

### Combined PI3Kδ and LAG3-blockade elicits superior tumor control

**Figure 6:**
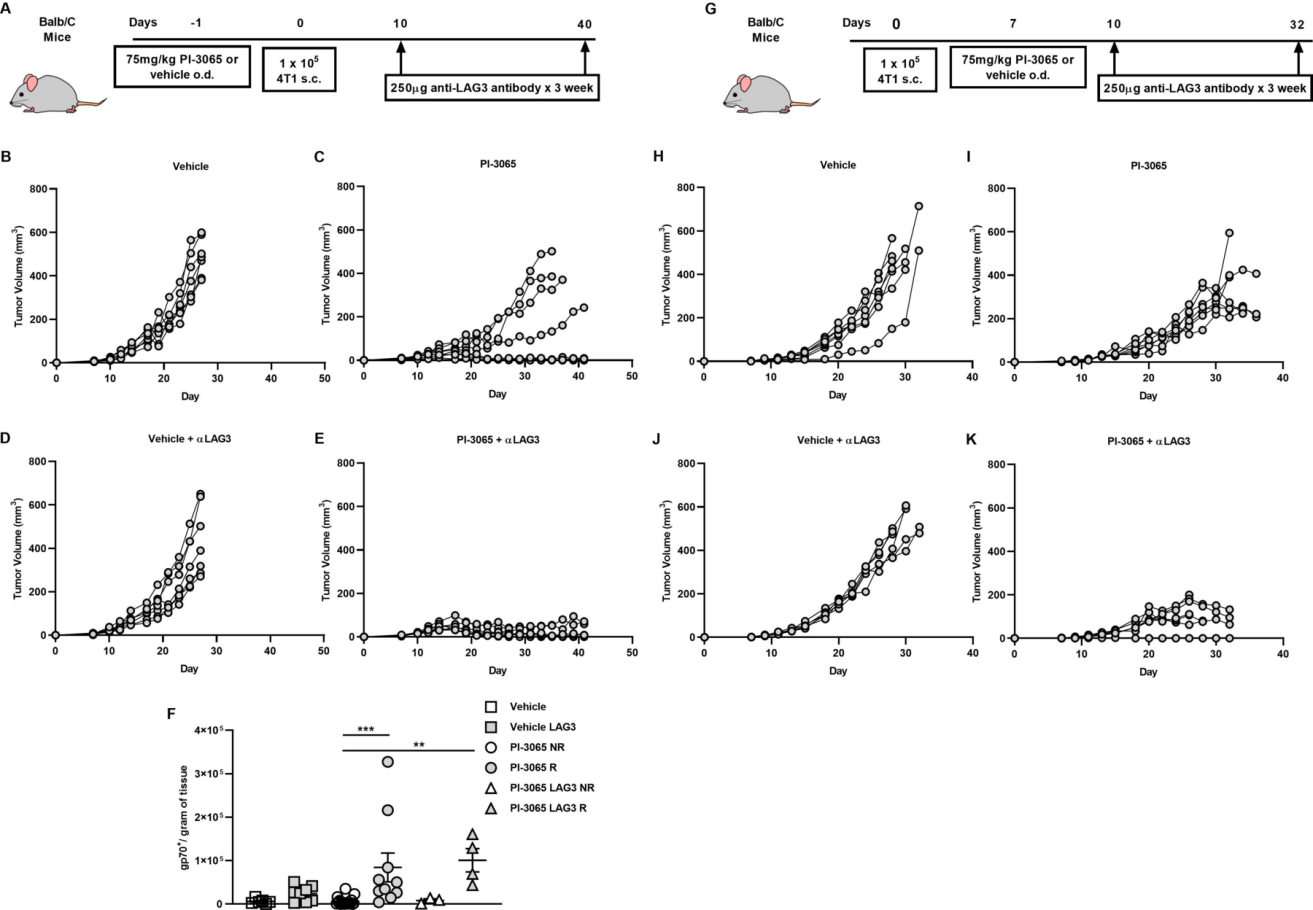
Anti-LAG3 and PI3Kδ combination therapy significantly reduces tumor burden in 4 T1 cells. (A) Schematic diagram showing the experimental protocol: BALB/c mice were treated daily for the duration of the study with either 75 mg/kg of PI-3065 or vehicle by oral gavage and were administered anti-LAG3 (250 µg) at the days indicated (6–8 mice/group). (B) Tumor growth curves from mice treated with (B) vehicle (C) PI-3065 (D) vehicle +anti-LAG3 or (E) PI-3065 +anti-LAG3 vehicle. (F) number of gp70 dextramer^+^ CD8^+^ T cells per gram of tumor tissue (NR: non-regressor, R; regressor). (G) Schematic diagram illustrating the experimental therapeutic strategy employed: BALB/c mice were inoculated with tumors and daily treatment with either 75 mg/kg of PI-3065 or vehicle by oral gavage commenced once tumors became palpable. Anti-LAG3 (250 µg) was administered at the days indicated (6–8 mice/group). Tumor growth curves from therapeutic strategy mice treated with (H) vehicle (I) PI-3065 (J) vehicle+anti-LAG3 or (K) PI-3065+anti-LAG3. All data are displayed as the mean±SEM statistical significance was Kruskal-Wallis test (**p≤0.01, ***p≤0.001).

As described earlier, a significantly higher proportion of Tregs expressing CD69 and particularly LAG3, distinguishes tumors that do not regress compared with those which do after treatment with PI-3065. Given that there was no significant difference in the number of Tregs in non-regressor versus regressor tumors, we considered it possible that the increase in expression of CD69 and/or LAG3, as shown in figure 5, was responsible for failure to control tumors by empowering the cells with greater suppressor capacity. To address this, we treated mice with both PI-3065 and either anti-CD69 or anti-LAG3 blocking antibodies. Previous studies have demonstrated that CD69-deficient mice or those treated with anti-CD69 antibody therapy have reduced tumor growth rate and metastatic burden,^35^ however, our study clearly demonstrates that anti-CD69 treatment alone or in combination with PI-3065 did not result in tumor control (online supplemental figure 1A and B). However, when anti-LAG3 blocking antibody was used in combination with PI-3065 this resulted in a significantly greater tumor control than either PI-3065 or anti-LAG3 treatment alone (figure 6B–E). Indeed, tumors regressed in 50% of the mice treated with the combination compared with mice treated with either PI-3065 alone (12%) or LAG3-blockade alone (0%) (figure 6C, D). Moreover, this was accompanied by a significant increase in the number of tumor-infiltrating AH1-specific CD8^+^ T cells (figure 6F).

10.1136/jitc-2020-000693.supp1 Supplementary data

**Figure 7:**
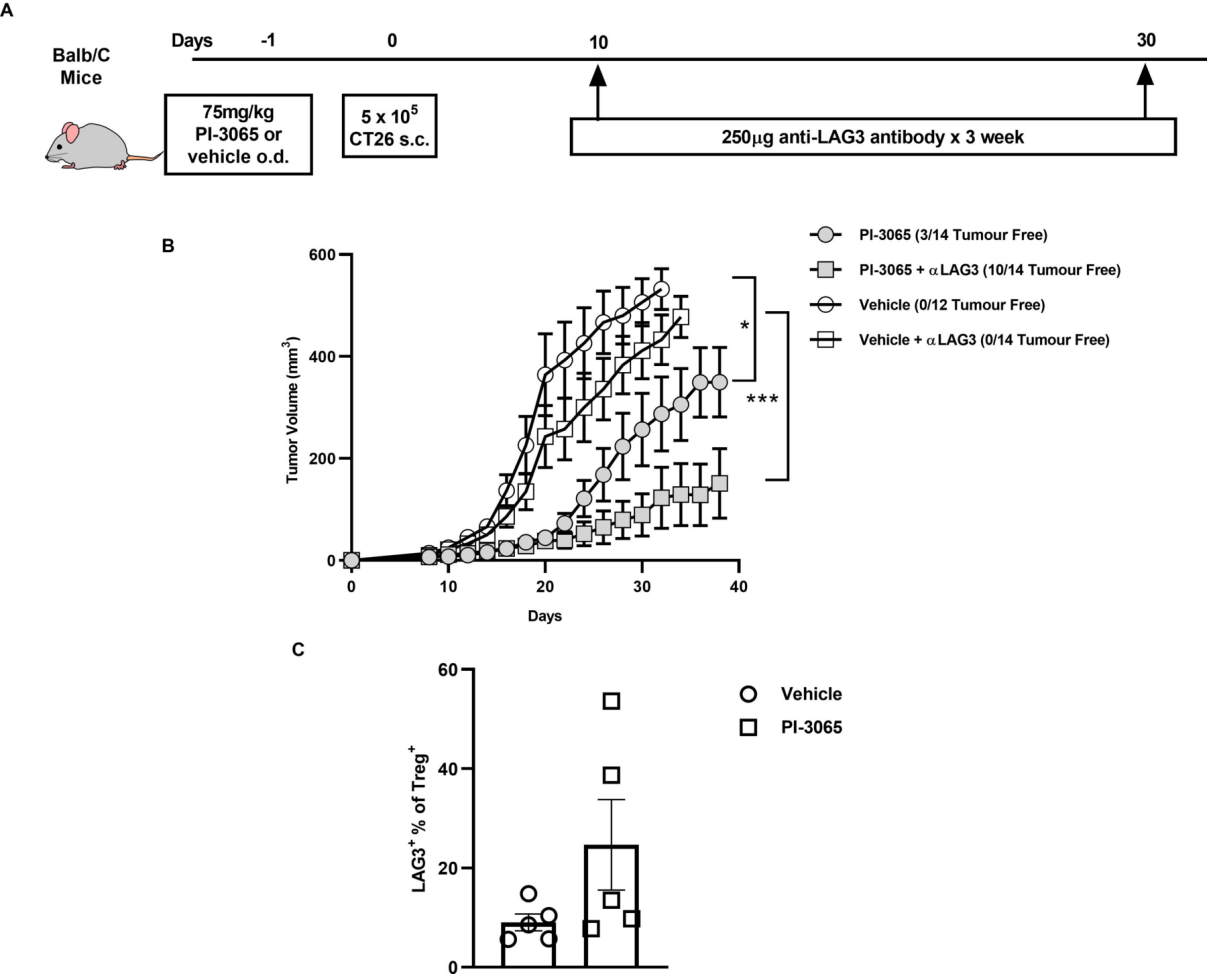
Anti-LAG3 and PI3Kδ combination therapy significantly reduces tumor burden in the CT26 model of colon adenocarcinoma. (A) Schematic diagram showing the experimental protocol: BALB/c mice were treated daily for the duration of the study with either 75 mg/kg of PI-3065 or vehicle by oral gavage and were administered anti-LAG3 (250 µg) at the days indicated (12–14 mice/group). (B) Tumor growth curves of mice inoculated with CT26 cells and treated with either vehicle, vehicle+anti-LAG3, PI-3065, or PI-3065+anti-LAG3. (C) Percentage of LAG3^+^ FoxP3^+^ Tregs in the tumors at day 24 post-tumor inoculation in the two treatment groups. All data are displayed as the mean±SEM. Statistical significance was determined by multiple unpaired t-test (*p≤0.05, ***p≤0.001).

We next sought to determine whether treatment with PI-3065 alone or in combination with LAG3-blockade, would enable control of other tumors, namely the colon carcinoma cell-lines, CT26 and MC38. We first found that while CT26 is responsive to PI-3065 treatment (figure 7A, B), the effect of PI-3065 treatment on the growth of MC-38 tumors was negligible (online supplemental figure 2 B & C). A comparison of TILs in each tumor type revealed that while the ratio of CD8^+^ T cells to Treg was markedly increased after treatment of 4T1 and CT26 tumors, this was not the case for MC38 tumors (8.51 and 4.84, respectively compared with 1.30; online supplemental figure 3), indicating that immunosuppression in MC38 tumors cannot be overcome by targeting PI3Kδ. As described earlier for 4T1 tumors, administration of anti-LAG3 antibodies had no effect on growth of either CT26 or MC-38 tumors when used alone. However, similar to the response in 4T1 tumors, combination therapy elicited superior tumor control of CT26 tumors while no effect was seen in the case of MC38 tumors (figure 7B, online supplemental figure 2 D & E). These data indicate that a robust response to the PI3Kδ-inhibitor, as observed for 4T1 and CT26 but not MC38 tumors, is an essential prerequisite to a therapeutic effect of anti-LAG3 antibodies.

10.1136/jitc-2020-000693.supp2 Supplementary data

10.1136/jitc-2020-000693.supp3 Supplementary data

LAG-3 blockade in combination with vehicle had no visible effect on the health of the mice. However, in the case of mice bearing 4T1 tumors, LAG3 blockade in combination with PI-3065 resulted in the development of varying degrees of autoimmunity with all animals developing a starry coat and inflammation of the skin a week after commencing dual treatment. Furthermore, in animals with slower growing but not regressing 4T1 tumors (50%), splenomegaly and lymphadenopathy was evident. Mice treated with PI-3065 only exhibited mild starring coat in the final week of treatment. No animal, in any of the treatment groups, displayed weight loss or signs of colitis.

Finally, to address the potential utility of PI3Kδ inhibition in therapeutic setting, we commenced PI-3065 treatment once tumors became palpable (day 7 post-tumor inoculation) either alone or in combination with anti-LAG3. While PI-3065 treatment alone significantly slowed tumor growth compared with vehicle (figure 6H, I), a delay in starting treatment did not result in any mice capable of regressing their tumors. However, when combination treatment with PI-3065 and anti-LAG3, as observed earlier, enhanced control of tumor growth, with clearance of tumors in some animals (figure 6J, K).

## Discussion

In 2014, Ali and colleagues used mice with genetically inactive PI3Kδ to demonstrate the requirement of this signaling protein for optimal Treg function. Mice bearing inactive PI3Kδ demonstrated enhanced tumor immunity; a finding which was recapitulated using the PI-3065 small molecule inhibitor of PI3Kδ.^6^ In an extension of this work, we have examined the impact of PI-3065 on Treg and intratumoral effector T cells identifying within the latter population, increased proliferation of antigen-specific T cells, enhanced self-renewal capacity, metabolic fitness and resistance to exhaustion as key consequences of successful PI3Kδ-targeted immunotherapy. Treatment with PI-3065 resulted in neogenesis of intratumoral HEV. Presence of HEV in tumors has been associated with a superior antitumor effector T cell response as a result of their ability to support migration of naïve and central memory T cells into the tumor. Thus, in the case of PI3Kδ-targeted immunotherapy, HEV may serve an important role in shaping the type and number of effector T cells entering the tumor mass.

Mice inoculated with 4T1 tumor cells and treated with PI-3065 could be divided into two groups, namely regressors and non-regressors. An interrogation of effector T cells revealed that a greater expansion of CD8^+^ T cells specific for the immunodominant 4T1 antigen, AH1,^38^ was observed in regressor compared with non-regressors and control tumors. Significant differences were also observed in the Treg populations. Treatment reduced Treg numbers in both tumor types, although the number of proliferating Treg was significantly lower in regressor tumors. Other key markers of Treg activity, namely CD69 and LAG3, also distinguished regressors and non-regressors. We have previously reported that highly proliferative, CD69^+^ Treg demonstrate superior suppressive capacity^37^; a finding which is in line with studies demonstrating that the enhanced suppressive function of CD69^+^ Treg significantly increases their ability to control autoimmune disease.^39^ However, while the proportion of Treg expressing CD69 is significantly lower in regressor compared with non-regressor 4T1 tumors, and despite previous reports,^35^ administration of CD69-specific antibodies in vivo did not improve tumor control in the 4T1 model. As mentioned earlier, the proportion of Treg expressing LAG3 was increased on treatment with PI-3065, particularly in non-regressor tumors. This may be explained by pre-existing LAG3^+^ Treg cells which, while avoiding the Treg-inhibitory effects of PI3Kδ-blockade, expand in response to the inflammation caused by PI-3065 administration. To target these cells, as well as LAG3^+^ CD8^+^ T cells, we determined whether LAG3-blocking antibodies would improve control of tumor growth. The results were striking showing in both 4T1 and CT26 tumor models, that the combination of PI3Kδ-blockade and anti-LAG3 antibodies significantly improved control of tumor growth. It is possible that PI3Kδ-blockade and anti-LAG3 antibodies both improve antitumor immunity indirectly through targeting Tregs and directly, through targeting effector T cells. It is clear however that not all tumors behave similarly. When we used the colon-derived cancer cell line MC-38, we observed a poor response to PI3Kδ-blockade, which could not be improved by the addition of LAG3-specific antibodies, suggesting other immunosuppressive mechanisms dominate in these tumors. Indeed, previous studies have demonstrated that MC38 tumors are far less susceptible to Treg inhibition than other tumor models, indicating that resistance to immunotherapy is provided by alternative cellular mechanisms.^40 41^ Furthermore, a study by Gyori and colleagues demonstrated that MC38 tumors were non-responsive to the PI3Kδ inhibitor, Idelalisib, and could only be controlled when therapies that targeted both Tregs and tumor-associated macrophages were employed together.^42^ It appears that an alteration in the CD8:Treg ratio is a requisite of successful PI3Kδ inhibition. In tumors, such as MC38, that fail to increase the CD8:Treg ratio following PI3Kδ inhibition, then second immunotherapies that target either Tregs or effector T cells are likely to prove ineffective. Our data point to the importance of assessing T cell subsets and phenotype where possible in order to identify tumor-specific bottlenecks, including those that arise in response to treatment. Accumulating this information will provide a framework to aid translational studies and guide selection of combination therapies. This will likely also be of key importance for the PI3Kδ inhibitors progressing in clinical trials in solid tumors, which include parsaclisib in combination with anti-PD1 in a range of advanced solid tumor indications (NCT02646748/NCT03589651), AMG-319 as monotherapy in a window-trial in Head and Neck cancer (Cancer Research UK and Amgen; NCT02540928) and IOA-244 (iOnctura) as monotherapy and in combination with pemetrexed/cisplatin in a range of advanced solid tumor indications (NCT04328844).

We further sought to improve the therapeutic relevance of our model by extending experiments to the treatment of mice with established 4T1 tumors. While PI3Kδ-blockade had some effect on control of established tumors, regression was observed on sequential administration of LAG3-specific antibodies, demonstrating the potential clinical utility of our findings.

Interestingly, the superior tumor immunity provided by PI-3065 and anti-LAG3 combination treatment was accompanied by autoimmunity. While this is a widespread finding in patients exhibiting successful tumor responses following immunotherapy with CTLA4-specific with or without PD1-specific antibodies, adverse inflammatory effects are not commonly found in mice treated in a similar way. The mouse model presented herein may therefore prove useful for probing the mechanisms underpinning concurrent autoimmunity and tumor immunity and for testing approaches for minimizing the former while maximizing the latter.

## Conclusions

This study demonstrates that treating mice with the PI-3065 small molecule inhibitor of PI3Kδ can promote control of tumor growth. A successful response is characterized by an increased population of TCF1^+^ CD69^-^ T cells that can self-renew, and continuously maintain a pool of effector cells that are able to efficiently use the available glucose and resist exhaustion. The study further identifies LAG3 as a critical checkpoint which diminishes the impact of PI3Kδ blockade, with combination treatment with PI-3065 and anti-LAG3 providing superior antitumor control over single agent therapy. Identification and sequential targeting of immune checkpoints provides an exciting route for combination therapy with PI3Kδ blockade.

## References

[1] Takeuchi Y, Nishikawa H Roles of regulatory T cells in cancer immunity. Int Immunol 2016;28:401–9. 10.1093/intimm/dxw025 27160722 PMC4986235

[2] Colbeck EJ, Jones E, Hindley JP, et al Treg depletion licenses T cell-driven HEV neogenesis and promotes tumor destruction. Cancer Immunol Res 2017;5:1005–15. 10.1158/2326-6066.CIR-17-0131 28947544 PMC5668144

[3] Scurr M, Pembroke T, Bloom A, et al Low-Dose cyclophosphamide induces antitumor T-cell responses, which associate with survival in metastatic colorectal cancer. Clin Cancer Res 2017;23:6771–80. 10.1158/1078-0432.CCR-17-0895 28855352 PMC5769815

[4] Scurr M, Pembroke T, Bloom A, et al Effect of modified vaccinia Ankara-5T4 and low-dose cyclophosphamide on antitumor immunity in metastatic colorectal cancer: a randomized clinical trial. JAMA Oncol 2017;3:e172579. 10.1001/jamaoncol.2017.2579 28880972 PMC5824319

[5] Gallimore A, Quezada SA, Roychoudhuri R Regulatory T cells in cancer: where are we now? Immunology 2019;157:187–9. 10.1111/imm.13088 31225653 PMC6587319

[6] Ali K, Soond DR, Pineiro R, et al Inactivation of PI(3)K p110δ breaks regulatory T-cell-mediated immune tolerance to cancer. Nature 2014;510:407–11. 10.1038/nature13444 24919154 PMC4501086

[7] Chellappa S, Kushekhar K, Munthe LA, et al The PI3K p110δ isoform inhibitor idelalisib preferentially inhibits human regulatory T cell function. J Immunol 2019;202:1397–405. 10.4049/jimmunol.1701703 30692213

[8] Patton DT, Garden OA, Pearce WP, et al Cutting edge: the phosphoinositide 3-kinase p110 delta is critical for the function of CD4+CD25+FoxP3+ regulatory T cells. J Immunol 2006;177:6598–602. 10.4049/jimmunol.177.10.6598 17082571

[9] Okkenhaug K, Graupera M, Vanhaesebroeck B Targeting PI3K in cancer: impact on tumor cells, their protective stroma, angiogenesis, and immunotherapy. Cancer Discov 2016;6:1090–105. 10.1158/2159-8290.CD-16-0716 27655435 PMC5293166

[10] Miller BW, Przepiorka D, de Claro RA, et al Fda approval: idelalisib monotherapy for the treatment of patients with follicular lymphoma and small lymphocytic lymphoma. Clin Cancer Res 2015;21:1525–9. 10.1158/1078-0432.CCR-14-2522 25645861

[11] Yang Q, Modi P, Newcomb T, et al Idelalisib: first-in-class PI3K delta inhibitor for the treatment of chronic lymphocytic leukemia, small lymphocytic leukemia, and follicular lymphoma. Clin Cancer Res 2015;21:1537–42. 10.1158/1078-0432.CCR-14-2034 25670221 PMC4523214

[12] Lim EL, Cugliandolo FM, Rosner DR, et al Phosphoinositide 3-kinase δ inhibition promotes antitumor responses but antagonizes checkpoint inhibitors. JCI Insight 2018;3. 10.1172/jci.insight.120626 PMC6124416 29875319

[13] Lim EL, Okkenhaug K Phosphoinositide 3-kinase δ is a regulatory T-cell target in cancer immunotherapy. Immunology 2019;157:210–8. 10.1111/imm.13082 31107985 PMC6587315

[14] Ahmad S, Abu-Eid R, Shrimali R, et al Differential PI3Kδ Signaling in CD4^+^ T-cell Subsets Enables Selective Targeting of T Regulatory Cells to Enhance Cancer Immunotherapy. Cancer Res 2017;77:1892–904. 10.1158/0008-5472.CAN-16-1839 28108509

[15] Dong S, Harrington BK, Hu EY, et al Pi3K p110δ inactivation antagonizes chronic lymphocytic leukemia and reverses T cell immune suppression. J Clin Invest 2019;129:122–36. 10.1172/JCI99386 30457982 PMC6307940

[16] Lin W-HW, Adams WC, Nish SA, et al Asymmetric PI3K signaling driving developmental and regenerative cell fate bifurcation. Cell Rep 2015;13:2203–18. 10.1016/j.celrep.2015.10.072 26628372 PMC4685001

[17] Nish SA, Zens KD, Kratchmarov R, et al Cd4+ T cell effector commitment coupled to self-renewal by asymmetric cell divisions. J Exp Med 2017;214:39–47. 10.1084/jem.20161046 27923906 PMC5206501

[18] Bowers JS, Majchrzak K, Nelson MH, et al PI3Kδ Inhibition Enhances the Antitumor Fitness of Adoptively Transferred CD8^+^ T Cells. Front Immunol 2017;8:1221. 10.3389/fimmu.2017.01221 29033940 PMC5626814

[19] Chen Y-H, Kratchmarov R, Lin W-HW, et al Asymmetric PI3K activity in lymphocytes organized by a PI3K-mediated polarity pathway. Cell Rep 2018;22:860–8. 10.1016/j.celrep.2017.12.087 29420173 PMC5806629

[20] Sade-Feldman M, Yizhak K, Bjorgaard SL, et al Defining T cell states associated with response to checkpoint immunotherapy in melanoma. Cell 2018;175:998–1013. 10.1016/j.cell.2018.10.038 30388456 PMC6641984

[21] Pulaski BA, Ostrand-Rosenberg S Mouse 4T1 breast tumor model. Curr Protoc Immunol 2001;Chapter 20:Unit 20.2. 10.1002/0471142735.im2002s39 18432775

[22] Carnevalli LS, Sinclair C, Taylor MA, et al PI3Kα/δ inhibition promotes anti-tumor immunity through direct enhancement of effector CD8^+^ T-cell activity. J Immunother Cancer 2018;6:158. 10.1186/s40425-018-0457-0 30587236 PMC6307194

[23] Gilchrist S, Gomes AL, Kinchesh P, et al An MRI-Compatible high frequency AC resistive heating system for Homeothermic maintenance in small animals. PLoS One 2016;11:e0164920. 10.1371/journal.pone.0164920 27806062 PMC5091850

[24] Kinchesh P, Gilchrist S, Beech JS, et al Prospective gating control for highly efficient cardio-respiratory synchronised short and constant TR MRI in the mouse. Magn Reson Imaging 2018;53:20–7. 10.1016/j.mri.2018.06.017 29964184 PMC6154312

[25] Okkenhaug K, Bilancio A, Farjot G, et al Impaired B and T cell antigen receptor signaling in p110delta PI 3-kinase mutant mice. Science (New York, NY 2002;297:1031–4. 10.1126/science.1073560 12130661

[26] Jones E, Gallimore A, Ager A Defining high endothelial venules and tertiary lymphoid structures in cancer. Methods Mol Biol 2018;1845:99–118. 10.1007/978-1-4939-8709-2_7 30141010

[27] Hindley JP, Jones E, Smart K, et al T-Cell trafficking facilitated by high endothelial venules is required for tumor control after regulatory T-cell depletion. Cancer Res 2012;72:5473–82. 10.1158/0008-5472.CAN-12-1912 22962270 PMC3491872

[28] O'Sullivan D, Sanin DE, Pearce EJ, et al Metabolic interventions in the immune response to cancer. Nat Rev Immunol 2019;19:324–35. 10.1038/s41577-019-0140-9 30820043

[29] Siska PJ, Beckermann KE, Mason FM, et al Mitochondrial dysregulation and glycolytic insufficiency functionally impair CD8 T cells infiltrating human renal cell carcinoma. JCI Insight 2017;2. 10.1172/jci.insight.93411. [Epub ahead of print: 15 Jun 2017]. PMC5470888 28614802

[30] Cretenet G, Clerc I, Matias M, et al Cell surface GLUT1 levels distinguish human CD4 and CD8 T lymphocyte subsets with distinct effector functions. Sci Rep 2016;6:24129. 10.1038/srep24129 27067254 PMC4828702

[31] Nguyen LT, Ohashi PS Clinical blockade of PD1 and LAG3--potential mechanisms of action. Nat Rev Immunol 2015;15:45–56. 10.1038/nri3790 25534622

[32] Turnis ME, Andrews LP, Vignali DAA Inhibitory receptors as targets for cancer immunotherapy. Eur J Immunol 2015;45:1892–905. 10.1002/eji.201344413 26018646 PMC4549156

[33] Scurr M, Ladell K, Besneux M, et al Highly prevalent colorectal cancer-infiltrating LAP⁺ Foxp3⁻ T cells exhibit more potent immunosuppressive activity than Foxp3⁺ regulatory T cells. Mucosal Immunol 2014;7:428–39. 10.1038/mi.2013.62 24064667 PMC3931584

[34] Esplugues E, Sancho D, Vega-Ramos J, et al Enhanced antitumor immunity in mice deficient in CD69. J Exp Med 2003;197:1093–106. 10.1084/jem.20021337 12732655 PMC2193974

[35] Mita Y, Kimura MY, Hayashizaki K, et al Crucial role of CD69 in anti-tumor immunity through regulating the exhaustion of tumor-infiltrating T cells. Int Immunol 2018;30:559–67. 10.1093/intimm/dxy050 30085193

[36] Yano H, Andrews LP, Workman CJ, et al Intratumoral regulatory T cells: markers, subsets and their impact on anti-tumor immunity. Immunology 2019;157:232–47. 10.1111/imm.13067 31087644 PMC6587321

[37] Colbeck EJ, Hindley JP, Smart K, et al Eliminating roles for T-bet and IL-2 but revealing superior activation and proliferation as mechanisms underpinning dominance of regulatory T cells in tumors. Oncotarget 2015;6:24649–59. 10.18632/oncotarget.5584 26433463 PMC4694785

[38] Huang AY, Gulden PH, Woods AS, et al The immunodominant major histocompatibility complex class I-restricted antigen of a murine colon tumor derives from an endogenous retroviral gene product. Proc Natl Acad Sci U S A 1996;93:9730–5. 10.1073/pnas.93.18.9730 8790399 PMC38497

[39] Cortés JR, Sánchez-Díaz R, Bovolenta ER, et al Maintenance of immune tolerance by Foxp3+ regulatory T cells requires CD69 expression. J Autoimmun 2014;55:51–62. 10.1016/j.jaut.2014.05.007 24934597 PMC4625610

[40] Teng MWL, Ngiow SF, von Scheidt B, et al Conditional regulatory T-cell depletion releases adaptive immunity preventing carcinogenesis and suppressing established tumor growth. Cancer Res 2010;70:7800–9. 10.1158/0008-5472.CAN-10-1681 20924111

[41] Liu J, Blake SJ, Harjunpää H, et al Assessing immune-related adverse events of efficacious combination immunotherapies in preclinical models of cancer. Cancer Res 2016;76:5288–301. 10.1158/0008-5472.CAN-16-0194 27503925

[42] Gyori D, Lim EL, Grant FM, et al Compensation between CSF1R+ macrophages and Foxp3+ Treg cells drives resistance to tumor immunotherapy. JCI Insight 2018;3. 10.1172/jci.insight.120631 PMC6124419 29875321

