# Supplementary material for: Enhanced antitumor immunity through sequential targeting of PI3Kδ and LAG3
Source: J Immunother Cancer. 2020 Oct 22;8(2):e000693. doi: 10.1136/jitc-2020-000693 (PMC7583804; doi:10.1136/jitc-2020-000693)
Supplement: Supplementary data [file jitc-2020-000693supp001.pdf]

Supplementary Figure 1

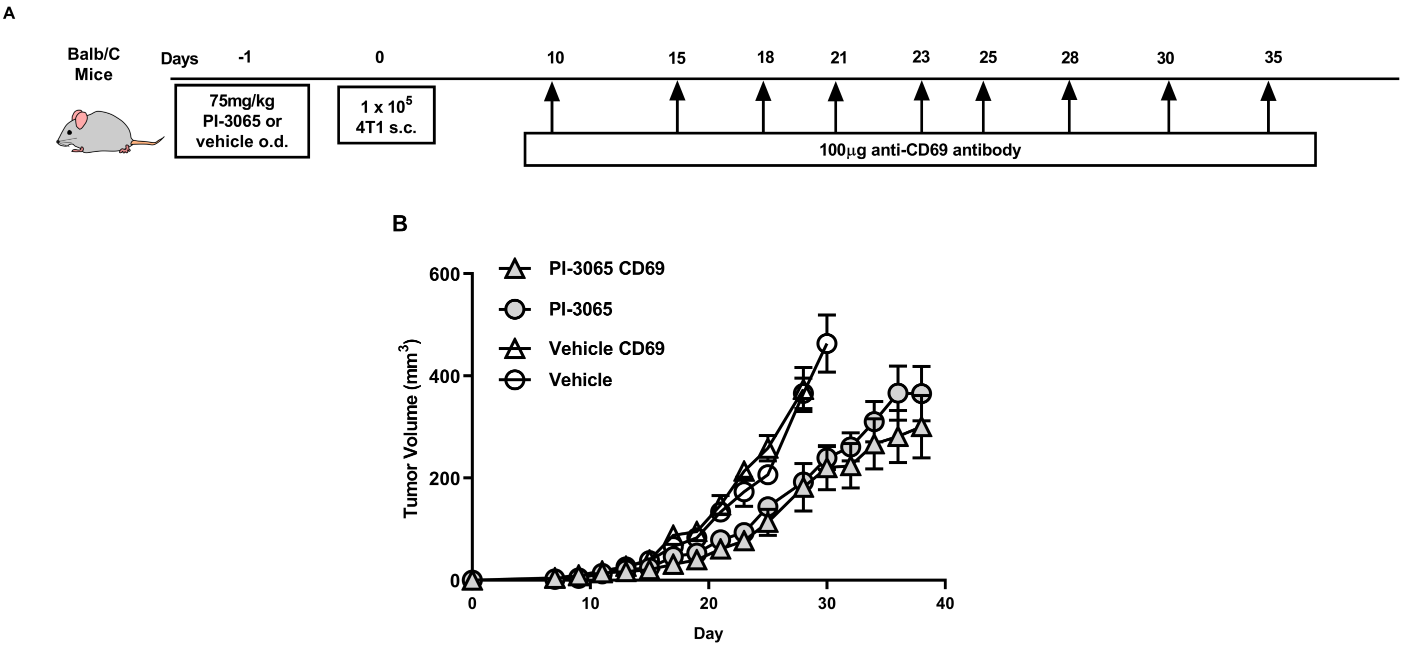

Supplementary Figure 1: anti-CD69 and PI3Kδ combination therapy does not alter tumor burden. (A) Schematic diagram shows the experimental protocol: Balb/C mice were treated daily for the duration of the study with either 75mg/kg of PI-3065 or vehicle by oral gavage and were administered anti-CD69 antibody (100μg) at the days indicated (8 mice/group). (B) Tumor growth curves from mice treated with PI-3065 or vehicle ± anti-CD69. All data is displayed as the mean±SEM.
