# Supplementary material for: Enhanced antitumor immunity through sequential targeting of PI3Kδ and LAG3
Source: J Immunother Cancer. 2020 Oct 22;8(2):e000693. doi: 10.1136/jitc-2020-000693 (PMC7583804; doi:10.1136/jitc-2020-000693)
Supplement: Supplementary data [file jitc-2020-000693supp002.pdf]

Supplementary Figure 2

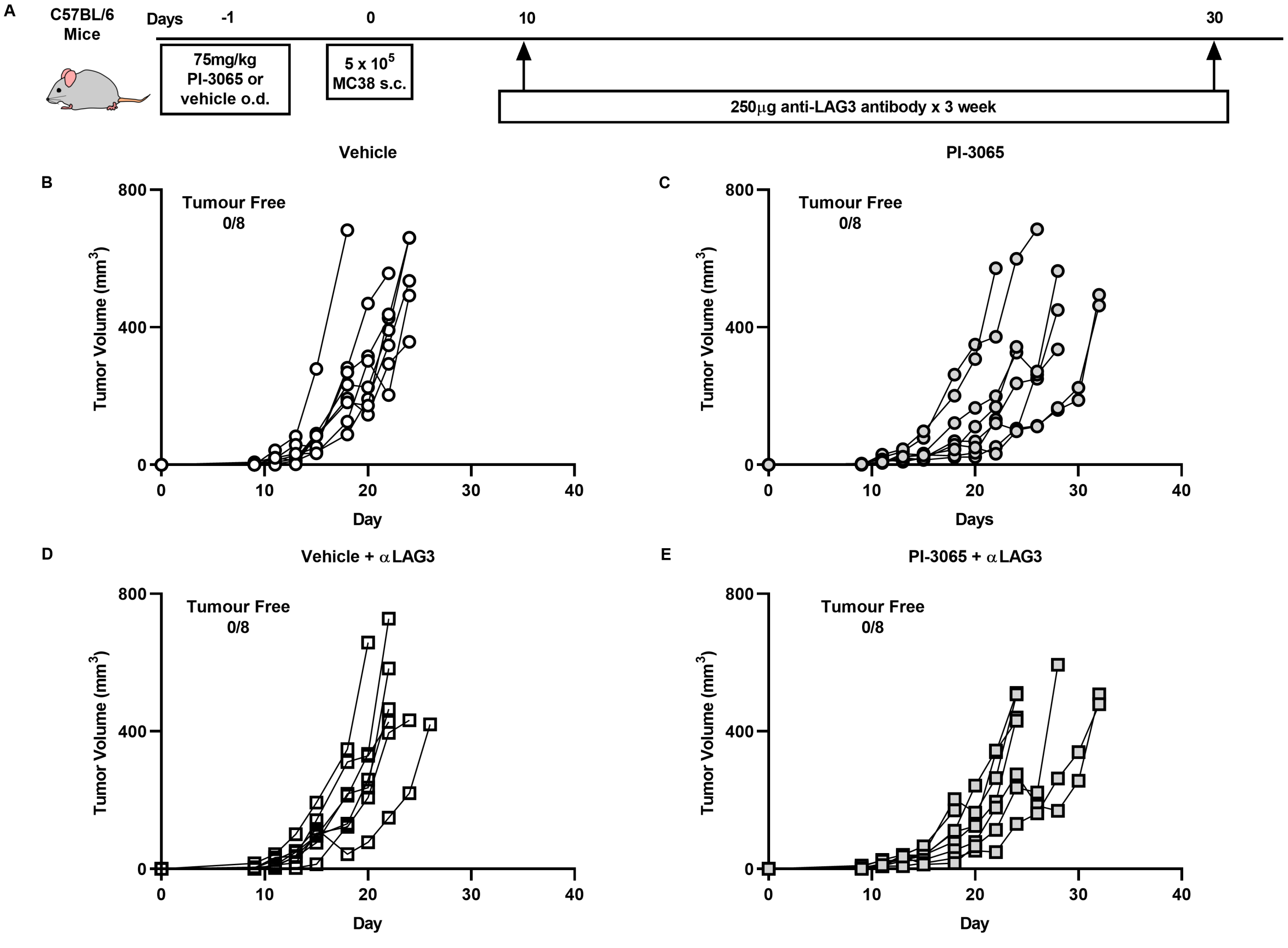

Supplementary Figure 2: Succesful PI3Kδ combination therapy is tumour model dependent. (A) Schematic diagram shows the experimental protocol: MC38 mice were treated daily for the duration of the study with either 75mg/kg of PI-3065 or vehicle by oral gavage and were administered anti-LAG3 (250μg) at the days indicated (8 mice/group). Individual growth curves from MC38 tumour bearing mice treated with (B) vehicle (C) PI-3065 (D) vehicle + anti-LAG3 or (E) PI-3065 + anti-LAG3.
