# Supplementary material for: Enhanced antitumor immunity through sequential targeting of PI3Kδ and LAG3
Source: J Immunother Cancer. 2020 Oct 22;8(2):e000693. doi: 10.1136/jitc-2020-000693 (PMC7583804; doi:10.1136/jitc-2020-000693)
Supplement: Supplementary data [file jitc-2020-000693supp003.pdf]

Supplementary Figure 3

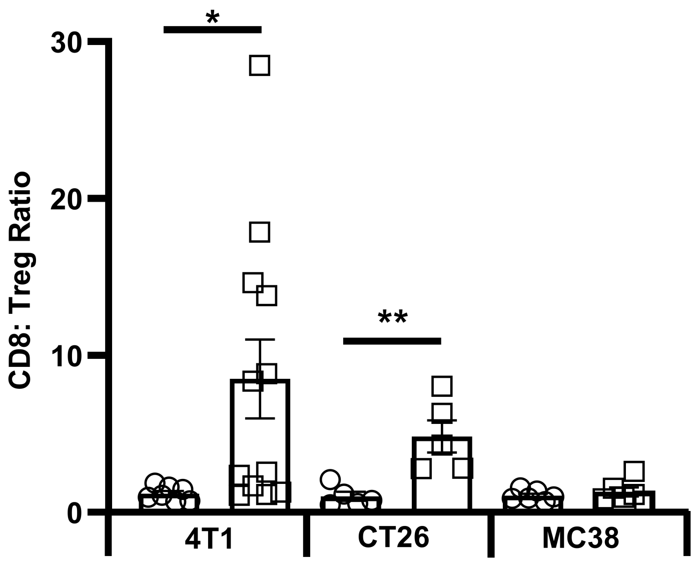

Supplementary Figure 3: High CD8:Treg ratio enables tumour control. The ratio of tumoural CD8:Tregs in the three tumour models. Data is displayed as the mean ± SEM (5 - 12 mice/group). Statistical significance was determined by unpaired T test.
